# Exploring the Potential Effects of Cryopreservation on the Biological Characteristics and Cardiomyogenic Differentiation of Rat Adipose-Derived Mesenchymal Stem Cells

**DOI:** 10.3390/ijms25189908

**Published:** 2024-09-13

**Authors:** Ahmed Farag, Sai Koung Ngeun, Masahiro Kaneda, Mohamed Aboubakr, Asmaa Elhaieg, Hanan Hendawy, Ryou Tanaka

**Affiliations:** 1 Faculty of Agriculture, Veterinary Teaching Hospital, Tokyo University of Agriculture and Technology, Tokyo 183-8509, Japan; 2 Department of Surgery, Anesthesiology, and Radiology, Faculty of Veterinary Medicine, Zagazig University, Zagazig 44519, Egypt; 3 Laboratory of Veterinary Diagnostic Imaging, Faculty of Agriculture, Tokyo University of Agriculture and Technology, Tokyo 183-8509, Japan; 4 Laboratory of Veterinary Anatomy, Division of Animal Life Science, Tokyo University of Agriculture and Technology, Tokyo 183-8509, Japan; 5 Department of Pharmacology, Faculty of Veterinary Medicine, Benha University, Toukh 13736, Egypt; 6 Department of Veterinary Surgery, Faculty of Veterinary Medicine, Suez Canal University, Ismailia 41522, Egypt

**Keywords:** cryopreservation, adipose-derived mesenchymal stem cells, cardiomyogenic differentiation, gene expression, cell viability

## Abstract

Cryopreservation is essential for the broad clinical application of mesenchymal stem cells (MSCs), yet its impact on their cellular characteristics and cardiomyogenic differentiation potential remains a critical concern in translational medicine. This study aimed to evaluate the effects of cryopreservation on the biological properties and cardiomyogenic capacity of rat adipose-derived MSCs (AD-MSCs). We examined their cellular morphology, surface marker expression (CD29, CD90, CD45), trilineage differentiation potential (adipogenic, osteogenic, chondrogenic), and gene expression profiles for the pluripotency marker *REX1* and immunomodulatory markers *TGFβ1* and *IL-6*. After inducing cardiomyocyte differentiation, we assessed cardiac-specific gene expressions (*Troponin I*, *MEF2c*, *GSK-3β*) using quantitative RT-qPCR, along with live/dead cell staining and immunofluorescence for cardiac-specific proteins (Troponin T, α-actinin, Myosin Heavy Chain). Cryopreserved AD-MSCs preserved their morphology, surface markers, and differentiation potential, but exhibited a reduced expression of *REX1*, *TGFβ1*, and *IL-6*. Additionally, cryopreservation diminished cardiomyogenic differentiation, as indicated by the lower levels of *Troponin I*, *MEF2c*, and *GSK-3β* seen compared to non-cryopreserved cells. Despite this, high cell viability (>90%) and maintained cardiac protein expression were observed post-cryopreservation. These findings highlight the necessity of optimizing cryopreservation protocols to ensure the full therapeutic potential of AD-MSCs, particularly in applications related to cardiac regenerative medicine.

## 1. Introduction

Cardiovascular diseases (CVDs) represent the foremost cause of death worldwide and are primarily attributed to the irreversible loss of functional cardiomyocytes [1,2,3]. In response to this challenge, numerous therapeutic strategies and materials have been investigated over the years [4,5]. Among these approaches, mesenchymal stem cells (MSCs) stand out as a particularly promising solution. MSCs are self-renewing, multipotent progenitor cells that have garnered significant interest in both scientific research and clinical applications due to their promising potential in regenerative medicine. [6]. MSCs facilitate tissue regeneration by releasing growth factors, cytokines, and extracellular vesicles, which together promote cell proliferation, inhibit apoptosis, and enhance the regenerative response [7]. Adipose-derived mesenchymal stem cells (AD-MSCs), in particular, hold significant potential in tissue engineering and regenerative medicine, largely due to their widespread availability and their ability to differentiate into a range of mesodermal tissues, such as bone, cartilage, and adipose tissue [8,9]. AD-MSCs can be cultured and expanded over long durations while retaining their ability to differentiate. This capability facilitates the production of substantial cell quantities, making them highly suitable for cell therapy applications. As a result, AD-MSCs are progressively being viewed as promising candidates for the replacement, repair, and regeneration of damaged or dead cells [10].

The future success of cell therapies depends significantly on efficient logistics and a robust delivery process. As Abazari et al. [11] emphasized, the clinical and logistical feasibility of delivering cell therapies is crucial to their effectiveness. Currently, cryopreservation is the industry standard for biopreservation [12], and the primary storage method for human MSC-based products [13]. Initially a peripheral process in cell therapy manufacturing, cryostorage has become essential for expanding the availability of stem cell therapies and regenerative medicine. However, despite its increasing importance, the field of cryobiology has not advanced as rapidly as the cell therapy industry [14].

Cryopreservation is crucial to the success of cell therapies for multiple reasons. It allows for the transport of cells, supports the establishment of cell banks with potentially unlimited shelf lives, guarantees a consistent supply of ready-to-use cells, and provides the necessary time for quality control testing and in vitro assays [12,15,16]. In numerous immunotherapy trials, the process of administering MSCs involves cryopreserving the cells, thawing them as required, and administering them within a few hours. This method is viable only if the thawed cells maintain their viability, safety, and efficacy [17]. Nevertheless, cryopreservation can lead to various types of physical and molecular damage, sparking debate over the effectiveness of fresh versus cryopreserved cells and whether cell viability necessarily equates to functional performance [18].

The conventional method of slow-rate freezing combined with rapid thawing using dimethyl sulfoxide (DMSO) as a cryoprotectant (CPA) has been widely employed for the cryopreservation of various stem cells, including murine and porcine embryonic stem cells [19,20,21]. However, when applied to human embryonic stem cells (hESCs), this approach often results in lower post-thaw survival rates, reduced plating efficiency, and a higher incidence of unwanted differentiation and a loss of pluripotency. These challenges are primarily attributed to ice crystal formation during freezing, which can disrupt cell–cell adhesion and compromise cell integrity [22,23,24]. Moreover, the quality of thawed cells can be significantly compromised compared to non-cryopreserved cells. For example, Francois et al. observed that freezing with DMSO altered the phenotype and proliferation of MSCs, with their post-thaw viability dropping significantly [25]. Similarly, Moll et al. reported that cryopreserved MSCs exhibited reduced therapeutic activity due to their increased proapoptotic properties and impaired immunosuppressive functions when compared to fresh cells [26].

To address these challenges, this study adopted Bambanker as the freezing medium, preserving cells at −80 °C, which provides an alternative to conventional methods [27,28]. For instance, Huang et al. successfully employed Bambanker for the cryopreservation of stem cells from adult dental pulp [29]. Unlike conventional methods that utilize fetal bovine serum (FBS) combined with DMSO, Bambanker contains bovine serum albumin (BSA) as its primary cryoprotective agent, which reduces the risk of xenogenic reactions post-transplantation [14,30]. The formulation of Bambanker allows for rapid cryopreservation at −80 °C without the need for a freezer program or pre-freezing steps, offering a practical and efficient alternative to traditional cryopreservation methods [27,31].

It is essential that cryopreserved MSCs retain their functional properties, such as their immunomodulatory effects and multilineage differentiation potential. A comprehensive biosafety assessment of these cryopreserved MSCs is critical prior to their use in clinical applications. Therefore, in this study, we aimed to comprehensively assess the effects of cryopreservation on rat AD-MSCs, focusing on their biological characteristics and cardiomyogenic differentiation. We hypothesized that using Bambanker for cryopreservation would minimize physical and molecular damage while preserving the viability and functionality of MSCs.

## 2. Results

### 2.1. Isolation and Morphology of Rat AD-MSCs

**Figure 1:**
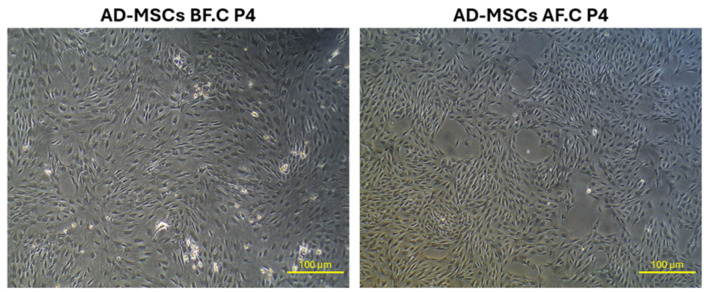
Morphological characteristics of plastic-adherent rat adipose-derived mesenchymal stem cells (AD-MSCs) at their fourth passage, both prior to and following cryopreservation. Abbreviations: AD-MSCs, adipose-derived mesenchymal stem cells; BF, before cryopreservation; AF, after cryopreservation; P4, passage 4. The scale bar represents 100 μm.

The morphological evaluation of AD-MSCs revealed distinct characteristics in both studied groups. AD-MSCs before cryopreservation (AD-MSCs BF.C.) displayed their typical spindle-shaped, fibroblast-like morphology with well-defined boundaries, indicating their healthy and undifferentiated state. These cells adhered uniformly to the culture dish surface, forming a consistent monolayer with robust confluency. Following cryopreservation (AD-MSCs AF.C.), the AD-MSCs maintained the characteristic spindle-shaped morphology of the non-cryopreserved group, showing no significant changes in their appearance before and after cryopreservation (Figure 1).

### 2.2. Surface Marker Expression of MSCs, Determined Using Flow Cytometry

**Figure 2:**
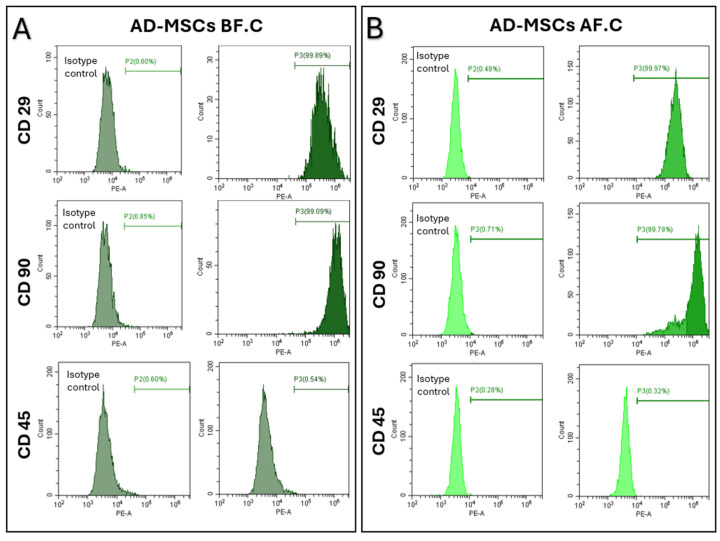
Representative histograms illustrating the expression of MSC surface markers (CD29 and CD90) and a hematopoietic marker (CD45). (**A**) Samples prior to cryopreservation. (**B**) Samples following cryopreservation.

The surface marker expression profiles of the AD-MSCs were assessed using flow cytometry, focusing on CD29 and CD90 as markers for mesenchymal stem cells, and CD45 as a marker for hematopoietic cells. Before cryopreservation (AD-MSCs BF.C.), the AD-MSCs exhibited a robust expression of CD29 and CD90, with high percentages of cells positive for CD29 (99.56 ± 0.32) and CD90 (99.36 ± 0.42), confirming the purity of the cell population. The expression of CD45, a marker for hematopoietic cells, remained consistently low or absent (0.22 ± 0.13), verifying the non-hematopoietic nature of AD-MSCs. After cryopreservation (AD-MSCs AF.C.), the AD-MSCs maintained similar expression profiles of CD29 (99.44 ± 0.25) and CD90 (99.46 ± 0.50), with a negligible CD45 expression (0.30 ± 0.1), consistent with their non-hematopoietic identity. These results indicate that cryopreservation preserved the surface marker expression profiles of AD-MSCs, confirming the maintenance of their MSC identity post-cryopreservation, without significant variation (Figure 2).

### 2.3. Differentiation Potential of Rat AD-MSCs

**Figure 3:**
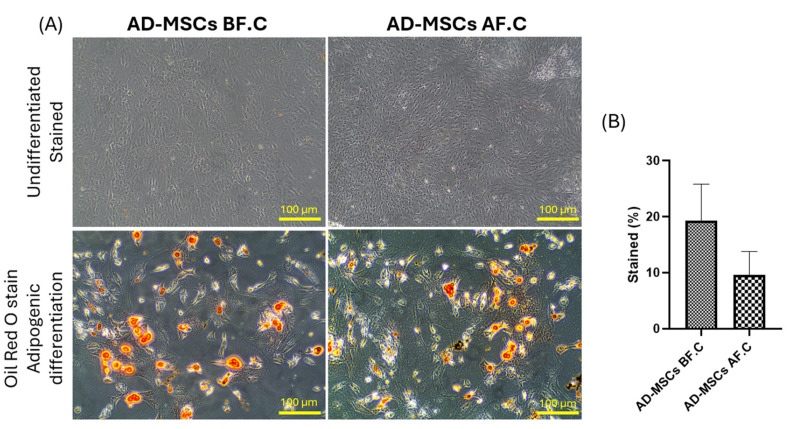
Adipogenic differentiation of AD-MSCs. (**A**) Representative histological images of both undifferentiated and Oil Red O-stained differentiated cells from rat AD-MSCs, before and after cryopreservation. (**B**) The percentage of Oil Red O-stained fat vacuoles was quantified using ImageJ (1.8.0-345). The scale bar represents 100 µm.

**Figure 4:**
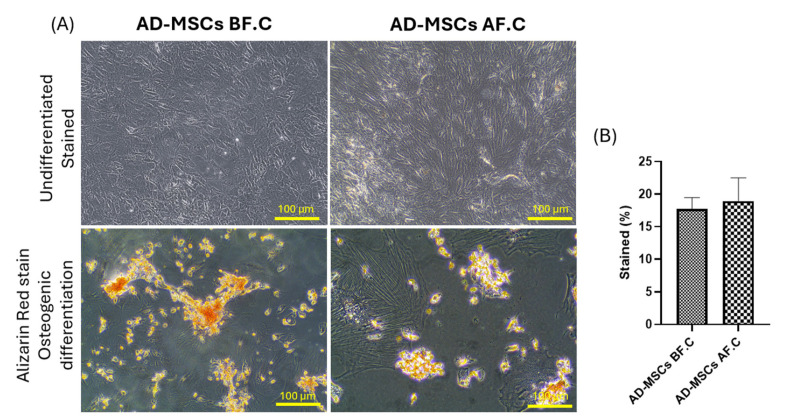
Osteogenic differentiation of AD-MSCs. (**A**) Representative histological images of both undifferentiated and Alizarin Red-stained osteogenic differentiated cells from rat AD-MSCs, before and after cryopreservation. (**B**) The percentage of Alizarin Red-stained mineral matrix deposition was quantified using ImageJ. The scale bar represents 100 µm.

**Figure 5:**
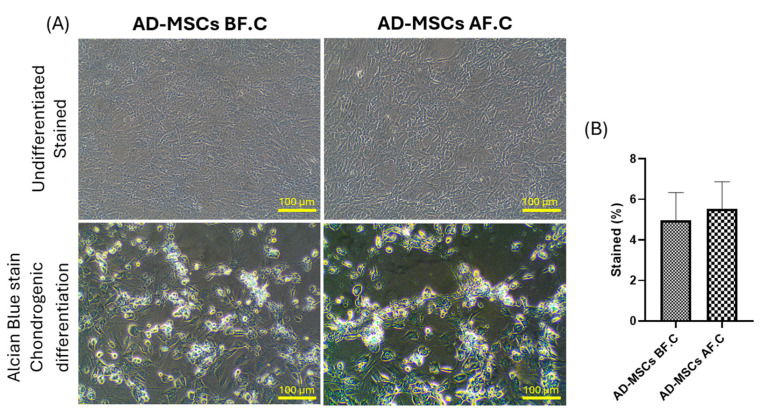
Chondrogenic differentiation of AD-MSCs. (**A**) Representative histological images showing both undifferentiated and Alcian Blue-stained chondrogenic differentiated cells from rat AD-MSCs, before and after cryopreservation. (**B**) The percentage of Alcian Blue-stained cartilage matrix was quantified using ImageJ. The scale bar represents 100 µm.

To assess the multipotentiality of rat AD-MSCs, the cells were exposed to specific induction media for adipogenic, osteogenic, and chondrogenic differentiation. Before cryopreservation (AD-MSCs BF.C.), the AD-MSCs demonstrated a robust differentiation potential across all tested lineages. Adipogenic differentiation resulted in the visible accumulation of lipid droplets stained with Oil Red O, osteogenic differentiation showed calcium deposition visualized by Alizarin Red staining, and chondrogenic differentiation exhibited glycosaminoglycan accumulation detected with Alcian Blue staining. Following cryopreservation (AD-MSCs AF.C.), the AD-MSCs maintained their differentiation capacity, with no significant difference in the percentage of stained cells observed between the two groups. However, their adipogenic differentiation showed a slight decrease in the number of cells accumulating lipid droplets compared to the non-cryopreserved group (9.57 ± 4.24 vs. 19.29 ± 6.51, *p* = 0.083), while their osteogenic and chondrogenic differentiation capabilities remained largely preserved (18.94 ± 3.57 vs. 17.73 ± 1.77, *p* = 0.628) and (5.54 ± 1.33 vs. 4.98 ± 1.36, *p* = 0.595), respectively (Figure 3, Figure 4 and Figure 5). These findings suggest that cryopreservation may minimally impact the adipogenic differentiation potential of AD-MSCs (*p* = 0.083), while their osteogenic and chondrogenic differentiation capabilities appear to be unaffected.

### 2.4. Gene Expression of Pluripotency and Immunomodulatory Markers

**Figure 6:**
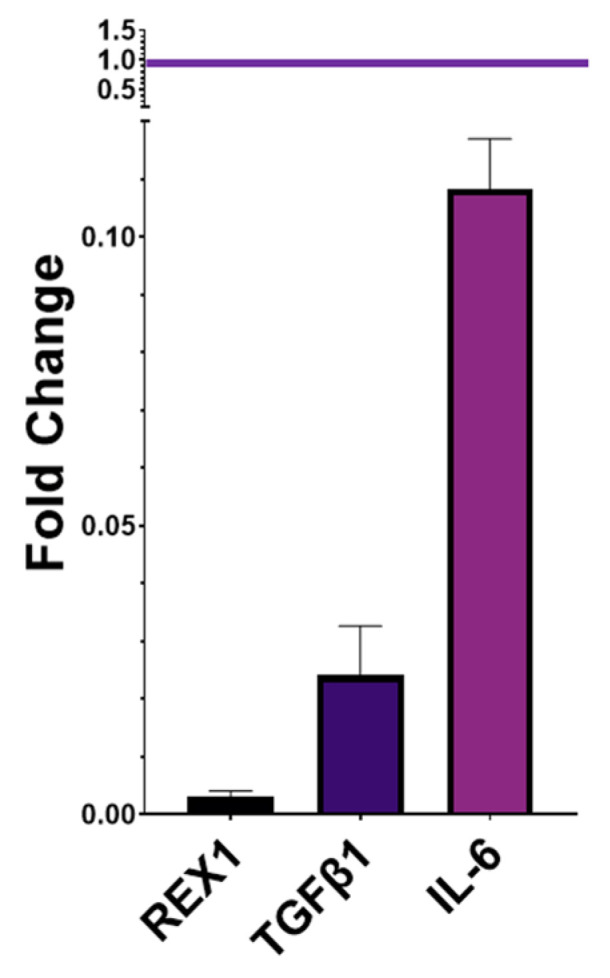
Bar graph representing the fold change expression levels of pluripotency and immunomodulatory markers in AD-MSCs after their cryopreservation, normalized with pre-cryopreservation AD-MSCs as the reference. All genes showed a decrease in their fold change, with values below 1. All experiments were conducted in triplicate to ensure the reliability of the results.

The gene expression profiles of *REX1*, *TGFβ1*, and *IL-6* were assessed in AD-MSCs before and after cryopreservation using the fold change method, with AD-MSCs before cryopreservation (AD-MSCs BF.C) serving as the reference. *REX1* exhibited a significant reduction after cryopreservation, with a fold change of 0.003 (*p* = 0.004). *TGFβ1* showed a fold change of 0.02 (*p* = 0.005). Similarly, *IL-6* demonstrated a fold change of 0.1 (*p* = 0.007). Overall, the expression levels of *REX1*, *TGFβ1*, and *IL-6* in AD-MSCs after their cryopreservation (AD-MSCs AF.C) were significantly reduced compared to those before cryopreservation (Figure 6).

### 2.5. Cardiomyocyte Differentiation and Morphology after Differentiation

**Figure 7:**
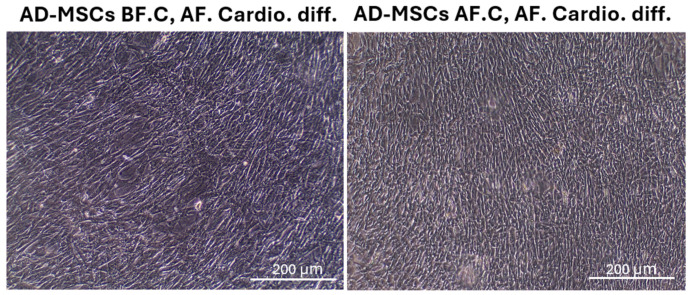
Morphological changes following cardiomyocyte differentiation in plastic-adherent rat AD-MSCs before and after cryopreservation. The scale bar represents 200 μm. Abbreviations: AD-MSCs, adipose-derived mesenchymal stem cells; BF, before cryopreservation; AF, after cryopreservation; AF. Cardio. Diff., after cardiomyocyte differentiation.

The AD-MSCs were subjected to a cardiomyocyte differentiation protocol, and their morphological changes were monitored using an inverted microscope. In the AD-MSCs before cryopreservation (AD-MSCs BF.C, AF. Cardio. diff.), the cells successfully differentiated into cardiomyocytes, displaying the expected elongated and striated morphology. These cells showed a high degree of homogeneity in their morphological characteristics, suggesting efficient differentiation. In the AD-MSCs after cryopreservation (AD-MSCs AF.C, AF. Cardio. diff.), the cells also differentiated into cardiomyocytes. However, there were minor differences in their morphology compared to the non-cryopreserved group. The cryopreserved AD-MSC-derived cardiomyocytes exhibited slightly less pronounced striations and fewer rod-shaped cells (Figure 7).

### 2.6. Cell Viability

**Figure 8:**
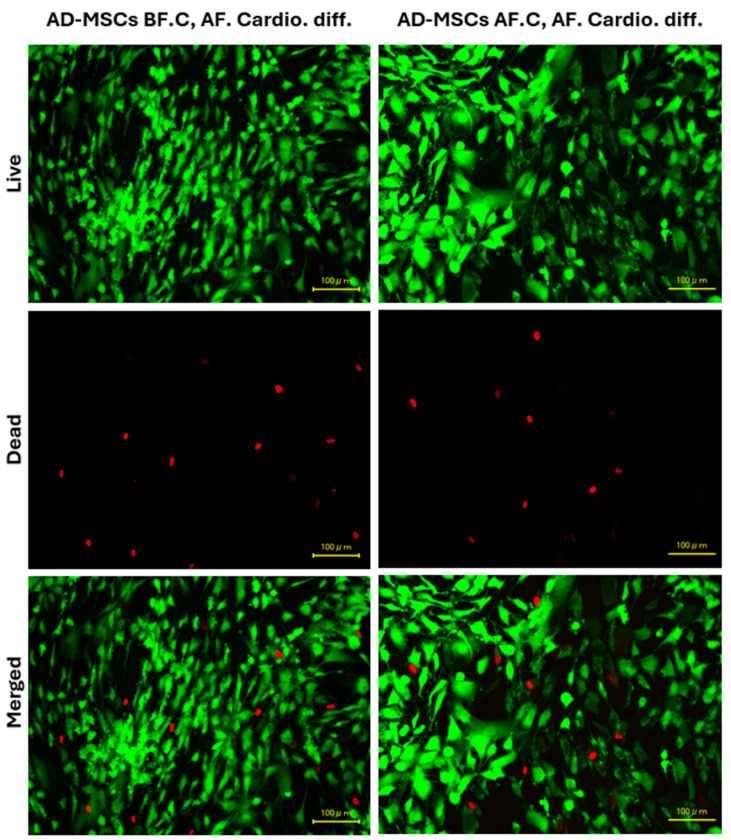
Viability of AD-MSCs assessed by live/dead (green/red) cell staining before and after cryopreservation. The scale bar represents 100 μm.

The viability of AD-MSCs before and after cryopreservation was assessed using the Live/Dead Cell Viability Assay Kit, which employs a two-color fluorescence method. Intense green fluorescence was observed in nearly all cell cytoplasms, indicating high cell viability in both AD-MSCs differentiated into cardiomyocytes before cryopreservation (AD-MSCs BF.C, AF. Cardio. diff.) and after cryopreservation (AD-MSCs AF.C, AF. Cardio. diff.). Our results show that the viability was 91.64 ± 1.471% before cryopreservation and 90.55 ± 1.164% after cryopreservation. The red staining of nuclei was seen in only a few cells in both groups, suggesting minimal cell death. There was no observable change in the number of dead cells before and after cryopreservation, indicating that cell viability was well retained post-cryopreservation (Figure 8). These results demonstrate that the cryopreservation process did not significantly impact the viability of AD-MSCs, as evidenced by the consistent presence of intense green fluorescence and minimal red staining in both groups.

### 2.7. Expression of Cardiac-Specific Proteins by Immunofluorescence Staining 

**Figure 9:**
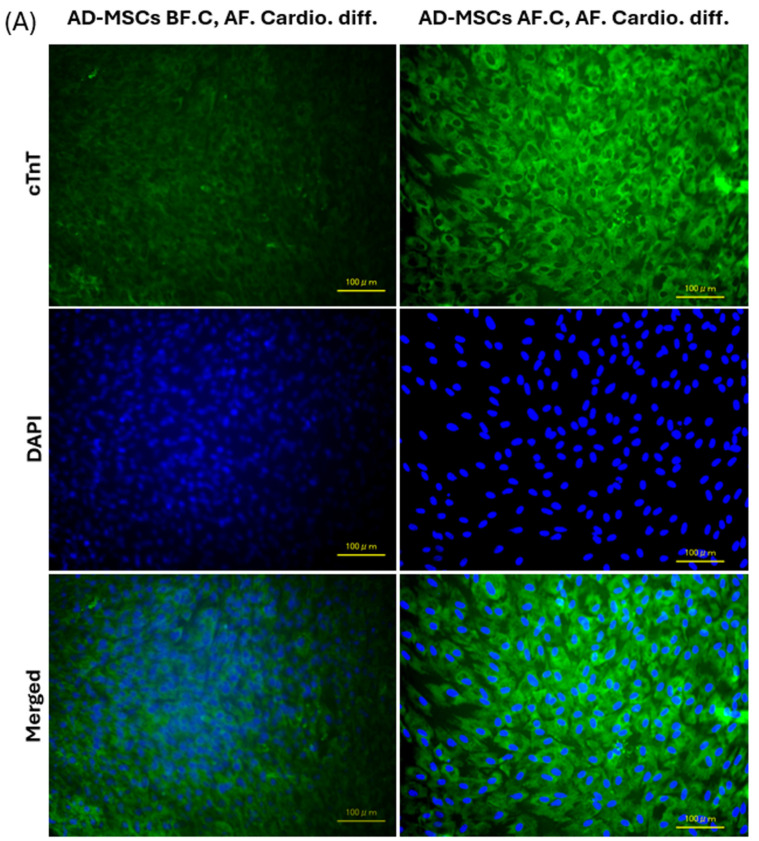

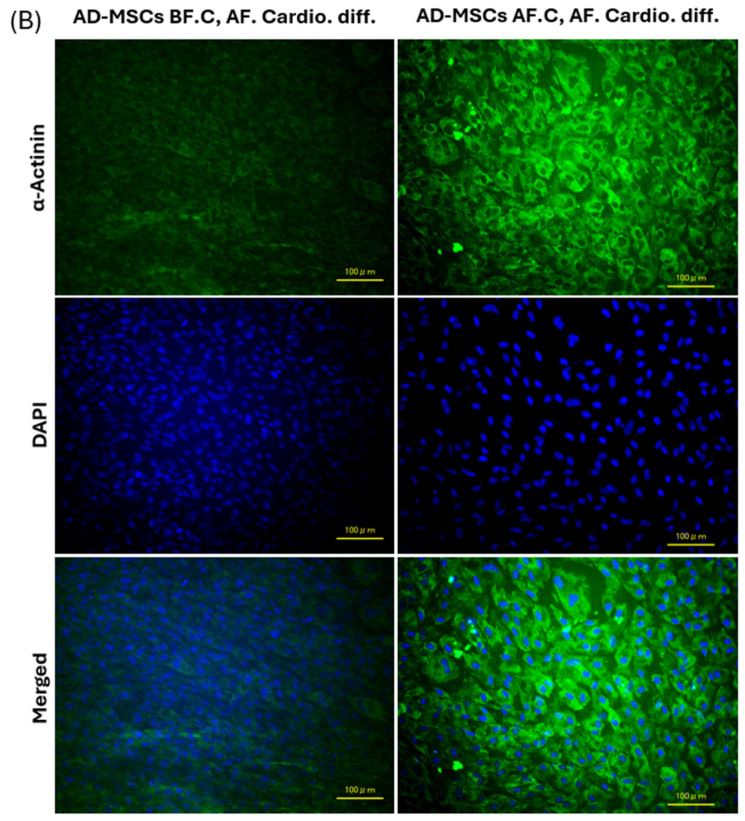

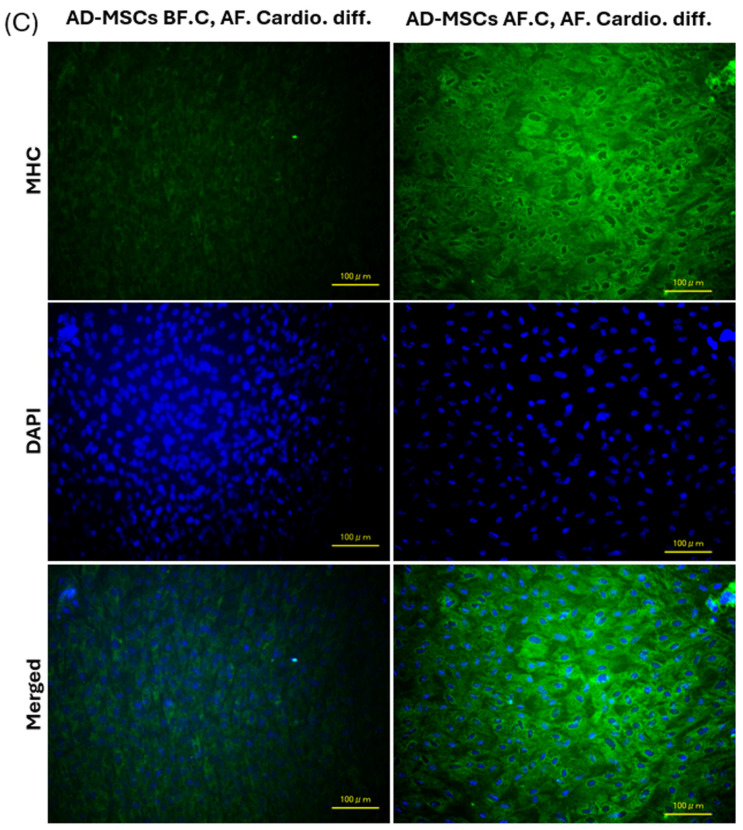
Immunofluorescence staining of cardiac-specific proteins in rat AD-MSCs before and after cryopreservation: (**A**) Troponin T, (**B**) α-Actinin, and (**C**) Myosin Heavy Chain 1. All experiments were conducted in triplicate to ensure the reliability of the results. The scale bar represents 100 μm.

Immunofluorescence staining was performed to evaluate the expression of cardiac-specific proteins—Troponin T, α-actinin, and Myosin Heavy Chain 1 (MHC)—in adipose-derived mesenchymal stem cells (AD-MSCs) before and after cryopreservation. Troponin T staining demonstrated a robust expression of this protein in both the non-cryopreserved (AD-MSCs BF.C, AF. Cardio. diff.) and cryopreserved (AD-MSCs AF.C, AF. Cardio. diff.) AD-MSC-derived cardiomyocytes. Characteristic striations typical of mature cardiomyocytes were evident in both groups. However, a notable increase in fluorescence intensity was observed in the cryopreserved group. Despite this, the overall spatial distribution and localization of Troponin T remained consistent between the two groups, suggesting that the fundamental expression pattern of Troponin T was preserved (Figure 9A).

Similarly, α-actinin staining confirmed the presence of organized sarcomeric structures in both groups, reflecting the maturation of the cardiomyocytes. Although there was a slight increase in fluorescence intensity in the cryopreserved group, the sarcomeric arrangement and overall expression pattern of α-actinin were comparable between the non-cryopreserved and cryopreserved groups, indicating their maintained structural integrity and functional characteristics (Figure 9B). MHC staining exhibited consistent patterns across the cell membranes in both groups. The cryopreserved cells showed higher fluorescence intensity, but their staining patterns were similar, suggesting that the structural integrity and functionality of the myocytes were preserved post-cryopreservation (Figure 9C).

In summary, while the fluorescence intensity of Troponin T, α-actinin, and MHC was higher in cryopreserved AD-MSC-derived cardiomyocytes, reflecting increased protein accumulation, the fundamental expression patterns and spatial distributions of these cardiac-specific proteins were consistent before and after cryopreservation. This indicates that the core expression profiles of these markers were maintained despite the observed differences in fluorescence intensity.

### 2.8. Quantitative Analysis of Cardiac-Specific Genes

**Figure 10:**
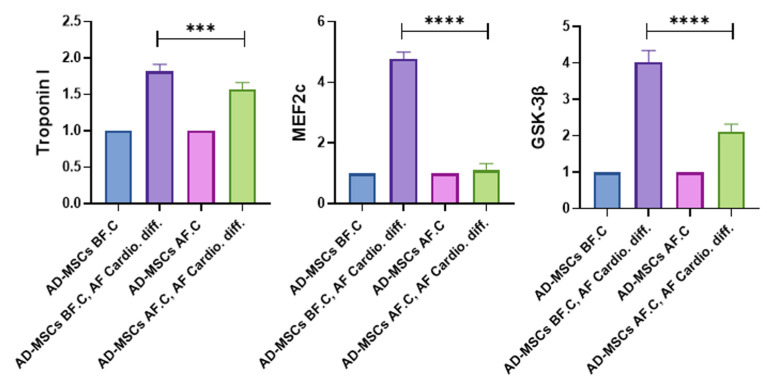
Quantitative analysis of cardiac-specific gene expression in rat AD-MSCs before (BF) and after (AF) cryopreservation using RT-qPCR. The expression levels of *Troponin I*, *MEF2c*, and *GSK-3β* are presented for the various study groups. All experiments were conducted in triplicate to ensure the reliability of the results. Statistical significance is indicated as follows: *** *p*  <  0.001 and **** *p*  <  0.0001.

The expression levels of cardiomyocyte-specific genes—*Troponin I*, Myocyte Enhancer Factor 2C (*MEF2c*), and Glycogen Synthase Kinase-3 beta (*GSK-3β*)—were quantitatively assessed using a fold change analysis (Figure 10). AD-MSCs before cryopreservation (AD-MSCs BF.C) and after cryopreservation (AD-MSCs AF.C) served as the control groups. In the AD-MSCs BF.C, AF. Cardio. diff. group, *Troponin I* expression increased by 1.84-fold, while in the AD-MSCs AF.C, AF. Cardio. diff. group, it increased by 1.6-fold. The upregulation in the AD-MSCs BF.C, AF. Cardio. diff. group was significantly higher than in the AD-MSCs AF.C, AF. Cardio. diff. group (*p* < 0.001).

*MEF2c* expression was markedly upregulated in the AD-MSCs BF.C, AF. Cardio. diff. group, by 4.7-fold, whereas, in the AD-MSCs AF.C, AF. Cardio. diff. group, it increased by only 1.03-fold. The difference in *MEF2c* upregulation between the two differentiated groups was statistically significant (*p* < 0.0001). Similarly, *GSK-3β* expression was upregulated by 3.97-fold in the AD-MSCs BF.C, AF. Cardio. diff. group, while in the AD-MSCs AF.C, AF. Cardio. diff. group it increased by 2.11-fold. The upregulation of *GSK-3β* was significantly higher in the AD-MSCs BF.C, AF. Cardio. diff. group compared to the AD-MSCs AF.C, AF. Cardio. diff. group (*p* < 0.0001).

## 3. Discussion

The present study aimed to elucidate the effects of cryopreservation on the biological characteristics and cardiomyogenic differentiation potential of rat AD-MSCs. As the clinical application of stem cells relies heavily on the preservation of their viability, functionality, and differentiation capacity, understanding these impacts is crucial. Our findings provide comprehensive insights into how cryopreservation affects various aspects of AD-MSCs, including their morphology, surface marker expression, trilineage differentiation potential, gene expression profiles, and ability to differentiate into cardiomyocytes.

In this study, we utilized a −80 °C cryopreservation method that uses Bambanker solution, differing from the conventional practice of storing cells in liquid nitrogen with dimethyl sulfoxide (DMSO) as a cryoprotectant. While DMSO is effective in preventing ice crystal formation, it is also known to have cytotoxic effects and can influence cell behavior post-thawing [32]. Previous studies have highlighted that DMSO can cause alterations in gene expression, induce apoptosis, and potentially affect the differentiation capacity of stem cells, making it imperative to explore alternative cryopreservation methods that minimize such risks [33]. By opting for a −80 °C storage method, our aim was to assess the viability and differentiation potential of MSCs under a less commonly used but potentially safer cryopreservation condition, thus contributing to the ongoing discussion regarding the optimization of stem cell storage practices.

The comparison between the −80 °C and liquid nitrogen storage methods reveals interesting insights. Although liquid nitrogen provides a near-perfect preservation environment by maintaining cells at temperatures that completely halt biological activity, these extreme conditions can also pose challenges such as mechanical stress and the need for specialized equipment [34]. In contrast, −80 °C storage is more accessible and less resource-intensive, but it may not prevent the formation of ice as effectively, potentially leading to reduced cell viability [35]. Despite these differences, our results demonstrated that MSCs preserved at −80 °C retained their ability to differentiate into cardiomyocytes, albeit with some variations in efficiency compared to previous reports that used liquid nitrogen storage [14]. These findings suggest that while −80 °C cryopreservation may not fully replicate the efficacy of liquid nitrogen storage, it offers a viable alternative that warrants further investigation, particularly in contexts where the use of DMSO is undesirable or where resource constraints limit access to liquid nitrogen facilities.

The phenotype of MSCs can be characterized by their morphology and surface marker (CD marker) expression. In this study, freshly cultured AD-MSCs displayed a typical spindle-shaped, fibroblast-like morphology with well-defined boundaries, aligning with previous reports [36,37]. Upon cryopreservation, AD-MSCs retained their characteristic mesenchymal stem cell morphology without significant changes, consistent with findings from other studies, which have reported no morphological alterations in MSCs post-cryopreservation [38,39,40]. However, some studies have observed variations in cell shapes at days 2 and 5 post-thaw and reported cell shrinkage detected via flow cytometry, indicating that cryopreservation may induce subtle morphological changes in certain contexts [26,41].

Similarly, our results demonstrated that the AD-MSCs, before cryopreservation, expressed mesenchymal-associated markers CD29 and CD90 while lacking the hematopoietic-associated marker CD45, in line with previous findings [42,43]. Post-cryopreservation, the AD-MSCs maintained consistent expression levels of the CD markers, indicating that their phenotypic profile remained largely unaffected by the cryopreservation process. This stability in surface marker expression aligns with several studies that have reported similar findings, suggesting that cryopreservation preserves the fundamental characteristics of MSCs [39,40,44,45,46,47].

To assess the multipotentiality of rat AD-MSCs, the cells were subjected to specific induction media for adipogenic, osteogenic, and chondrogenic differentiation. After cryopreservation, AD-MSCs retained their tri-lineage differentiation capacity, demonstrating a robust differentiation potential across all tested lineages. Adipogenic differentiation was indicated by the presence of lipid droplet accumulation, with cryopreservation showing no significant effect on this process. These results align with earlier research [48,49,50]. While the change was not statistically significant, our study revealed a minor reduction in the number of cells accumulating lipid droplets in the cryopreserved group compared to the non-cryopreserved group. This observation aligns with findings by Lauterboeck et al., who provided a quantitative assessment of adipogenesis and reported a lower differentiation level post-cryopreservation [51]. As for osteogenic differentiation, our study showed calcium deposition visualized by Alizarin Red staining, indicating that the cells’ osteogenic differentiation capabilities remained largely preserved following cryopreservation. These results are in line with other studies [46,52,53,54]. Similarly, their chondrogenic differentiation exhibited glycosaminoglycan accumulation detected by Alcian Blue staining, with no obvious effect after cryopreservation, indicating that their chondrogenic differentiation capabilities remained largely preserved. These findings are consistent with previous research [55,56]. James et al. found that adipose-derived MSCs preserved in a solution of 10% DMSO and 90% FBS exhibited reduced adipogenic potential compared to fresh cells. This decrease in their differentiation capacity was linked to the downregulation of genes associated with adipogenesis and osteogenesis [57]. Additionally, some studies have reported a lower osteogenesis capacity post-thaw, while others noted improved osteogenesis [58,59]. These discrepancies highlight the variability in differentiation potential outcomes based on specific cryopreservation protocols and conditions.

Stemness markers, including *REX1*, were utilized to assess the multipotency of stem cells [60,61]. Previous research has shown that MSCs display a positive expression of *OCT-4*, *REX1*, and *SOX-2* [62]. In this study, our findings indicate that cryopreservation significantly impacts pluripotency markers, as shown by the reduced expression of *REX1* in cryopreserved AD-MSCs. These results are consistent with those of Mamidi et al., who reported that stemness markers such as *OCT-4*, *NANOG*, *SOX-2*, and *REX1* were positively expressed in MSCs following their cryopreservation, suggesting their potential for multipotency. However, Mamidi et al. also observed that the levels of these markers decreased in later passages after repeated cryopreservation [55]. On the other hand, Yong et al. reported that human adipose-derived stem cells (ASCs) preserved through cryopreservation exhibited notably higher levels of stemness genes, such as *OCT-4*, *REX-1*, *SOX-2*, and *NANOG*, compared to their fresh counterparts. This indicates an improved ability to maintain their stemness characteristics. Despite these elevated marker levels, the cryopreserved ASCs continued to show normal proliferation and differentiation capabilities [63]. It is important to note, as highlighted by Choi et al. [61] and Pierantozzi et al. [64], that the improved proliferative and differentiation potential of MSCs is not always directly linked to the levels of their stemness markers. Instead, factors like oxygen tension and the culture environment, whether two-dimensional (2D) or three-dimensional (3D), have a more significant impact on MSCs’ proliferation and differentiation activity [61,65]. These observations underscore the complexity of MSC biology and the multifaceted nature of stemness and differentiation regulation in these cells.

Our results indicated that cryopreservation significantly affects the immunomodulatory markers in AD-MSCs, as evidenced by the downregulation of their gene expression levels. These results align with those of Moll et al., who observed that cryopreserved MSCs exhibit reduced immunomodulatory and blood regulatory functions immediately after thawing, leading to a faster complement-mediated clearance when exposed to blood [26]. Similarly, François et al. showed that cryopreservation negatively impacts the immunosuppressive properties of MSCs, though this effect is reversible. This alteration is linked to a heat-shock stress response induced during the thawing process [25]. These disparities with previous reports indicating the negative effects of cryopreservation and thawing on MSC performance can be attributed to variations in the tissues’ source and handling during the manufacturing process, including differences in cryopreservation and thawing techniques [17,66,67]. Certain studies propose that cryopreservation itself is not detrimental, and the variability in MSC activity reported previously may be attributed to variations in manufacturing techniques, including mechanical handling, expansion, freezing, thawing, and reconstitution. For instance, research involving xenogen-free human bone marrow-derived GMP-MSCs showed that cryopreservation did not impact the cells’ immunomodulatory capabilities in vitro [68].

In this study, we reset the passage number to 1 after cryopreservation to distinguish between the pre- and post-cryopreservation stages of AD-MSCs. This approach effectively placed the cells at passage 8 from their initial derivation but simulated a common scenario where cells are revived and cultured anew. While higher passage numbers can lead to senescence and reduced functionality, MSCs typically retain their multipotency and therapeutic potential up to passage 10 under optimal conditions [69,70]. We assessed cells at passage 4 post-thaw to reflect their practical applications, as early passages are often used to ensure quality and functionality [71]. Additionally, we allowed the cells to recover and undergo acclimatization after thawing to mitigate transient stress responses and regain their functional stability, aligning with research showing that MSCs can recover their activity within 24 h post-thaw [31,72].

Regarding the morphological changes in cardiomyocytes following their cryopreservation, our observations revealed that the cryopreserved cells maintained an intact structure with clear striations, comparable to freshly isolated cells. This suggests that the cryopreservation process did not adversely affect their cellular architecture. Similar findings were reported by Zhou et al. [73], reinforcing the notion that cryopreserved cardiomyocytes can retain their structural integrity. This preservation of morphology is critical for the functional viability of cardiomyocytes post-thaw and highlights the effectiveness of our cryopreservation protocol in maintaining cell quality.

Evaluating cell viability is essential to ensure the therapeutic efficacy of cryopreserved MSCs. Using the Live/Dead Cell Viability Assay, our results demonstrate that the cryopreservation process did not significantly impact the viability of AD-MSCs. These findings align with other studies that have reported no change in viability immediately after thawing [39,42,51,73]. This consistent viability could be attributed to Davies et al., who found that MSCs from adipose tissue are more robust and able to endure extended periods outside of culture compared to MSCs derived from bone marrow or dental pulp [45,74]. Adipose tissue is known to contain a subset of multi-lineage differentiating stress-enduring (Muse) cells, which possess the ability to withstand severe conditions such as hypoxia, a lack of serum, extended exposure to proteolytic enzymes like collagenase, and low temperatures [45,74]. In contrast, other studies have reported a decline in the viability of bone marrow cells (BMCs) after cryopreservation. This could be due to the relatively small percentage of stem cells present in bone marrow (0.001–0.1%) compared to the higher proportions in adipose tissue (6–10%) and dental pulp (61%) [75,76,77]. The decline in viability observed in the BMC population is probably linked to nucleated non-progenitor cells, which are generally more vulnerable to cryopreservation than MSCs [78]. These differences highlight the importance of the tissue’s source in cryopreservation outcomes and the relative robustness of adipose-derived MSCs.

To further characterize the cardiomyocytes, we examined the expression of key cardiomyocyte-associated proteins using immunofluorescence staining. Our analysis focused on cardiac troponin T (cTnT), α-actinin, and myosin heavy chain (MHC), which are integral to the function of cardiac muscle. cTnT is crucial for regulating cardiac muscle contraction and serves as a reliable marker of cardiomyocyte differentiation [79]. α-actinin is an actin-binding protein essential for the structural organization of the sarcomere in cardiomyocytes [80]. MHC is vital for the contraction and mechanical function of cardiac muscle [81]. Our results demonstrated a notable increase in the fluorescence intensity of these markers post-cryopreservation, suggesting an enhancement in protein accumulation. However, the spatial distribution and overall expression patterns of these proteins remained consistent between the pre- and post-cryopreservation samples.

The observed increase in fluorescence intensity post-cryopreservation could reflect several underlying phenomena. Cryopreservation and subsequent thawing processes induce cellular stress responses that can affect protein synthesis and turnover. This increased fluorescence intensity may indicate a temporary accumulation of cardiac-specific proteins as cells recover from cryopreservation-induced stress, rather than a change in the fundamental expression patterns of these proteins [31,82,83]. Such findings align with reports in the literature, which suggest that cryopreservation-induced stress results in altered protein dynamics, including variations in protein accumulation and localization [31,84,85]. Conversely, in non-cryopreserved AD-MSCs, the observed faint immunofluorescence staining for cardiac-specific proteins might be attributed to several factors. One possible explanation is that the staining intensity could be influenced by differences in antibody binding efficiency or the overall cellular antigenic landscape, which may vary between non-cryopreserved and cryopreserved cells [86]. Despite this, our results suggest that cryopreservation effectively preserves cardiac-specific proteins, consistent with the findings of Correia et al., who reported that human pluripotent stem cell-derived cardiomyocytes (hPSC-CMs) in three-dimensional aggregates exhibit an enhanced resistance to damage from prolonged hypothermic storage compared to those in 2D monolayers. Correia et al. found that these cells maintained their ultrastructure, gene and protein expression, electrophysiological characteristics, and drug responsiveness after storage, supporting the efficacy of advanced preservation methods in maintaining cell functionality [87]. Additionally, Brink and colleagues investigated the in vitro functional and molecular characteristics of fresh and cryopreserved human induced pluripotent stem cell-derived cardiomyocytes (hiPSC-CMs) from multiple independent lines. Their findings indicated that cryopreservation does not adversely affect the molecular, physiological, or mechanical properties of hiPSC-CMs and may even enhance the recovery of ventricular myocytes [88].

Our study reveals a differential impact of cryopreservation on the upregulation of cardiac-specific genes in AD-MSCs, with cryopreserved cells demonstrating lower expression levels of *Troponin I*, *MEF2c*, and *GSK-3β* compared to non-cryopreserved cells. *Troponin I*, a critical marker for cardiomyocyte function [79], exhibited a significant upregulation in non-cryopreserved AD-MSCs after their differentiation, whereas cryopreserved cells showed a comparatively modest increase. Similarly, *MEF2c* and *GSK-3β*, which are essential for cardiac muscle development and function [89,90], also displayed higher expressions in non-cryopreserved cells post-differentiation. These findings suggest that the cryopreservation process may attenuate the cardiomyogenic potential of AD-MSCs, potentially due to cellular stress or alterations in the transcriptional regulation mechanisms triggered during freezing and thawing. Our results are consistent with previous studies indicating that cryopreservation can affect the differentiation efficiency and functional gene expression of stem cells [14,91,92,93]. Therefore, while cryopreserved AD-MSCs retain their ability to differentiate into cardiomyocytes, their reduced gene expression levels highlight the need for optimized cryopreservation protocols to enhance the therapeutic efficacy of stem cell-based cardiac regenerative therapies.

To the best of our knowledge, this study marks the first comprehensive investigation into the impact of cryopreservation on rat AD-MSCs, utilizing a wide-ranging evaluation approach to explore both biological characteristics and cardiomyogenic differentiation. This preliminary research sets the stage for further detailed explorations of how cryopreservation specifically influences these cells, paving the way for deeper insights into optimizing their use in biomedical applications.

## 4. Materials and Methods

### 4.1. Design of Experimental Groups

This study involved five male Sprague Dawley rats, aged 8 to 10 weeks and weighing between 300 and 350 g. These rats were maintained in an environment-controlled setting, with temperatures set between 25 and 30 °C, humidity levels of 50 to 60%, and a 12-h light/dark cycle. They had continuous access to dry food and water. Euthanasia was carried out using an overdose of isoflurane administered through inhalation, as outlined by Siennicka et al. [94].

This study adhered to the protocols approved by the Animal Care and Use Committee at Tokyo University of Agriculture and Technology (approval number: R05-158).

**Figure 11:**
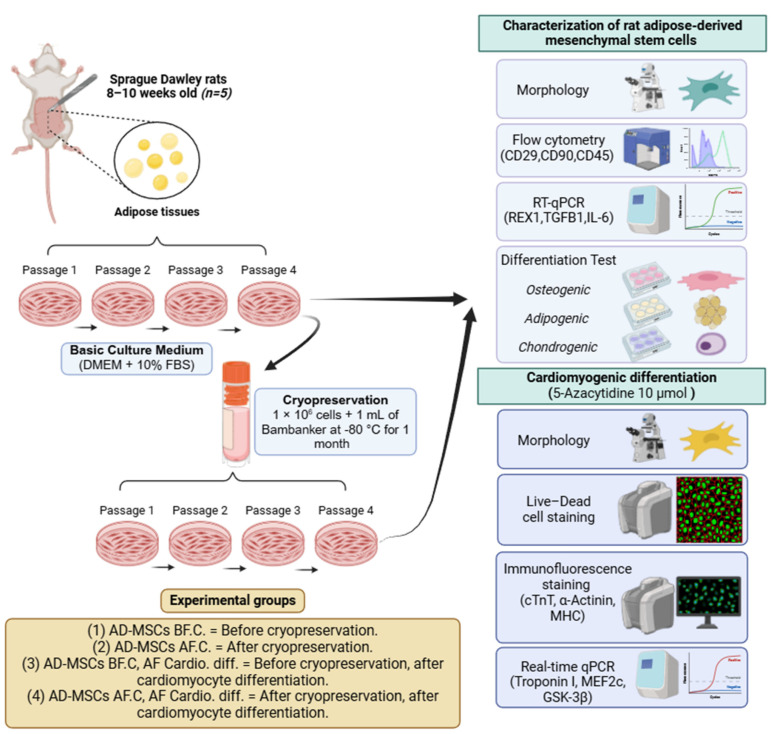
Overview of the study’s experimental design.

Cells obtained from passage 4 adipose tissue samples were identified as AD-MSCs. These cells were categorized into two main groups: AD-MSCs before cryopreservation (AD-MSCs BF.C) and AD-MSCs after cryopreservation (AD-MSCs AF.C). These groups served as control groups to evaluate cardiomyocyte differentiation both before and after cryopreservation. All experimental methods were conducted in triplicate to ensure reliable results. Figure 11 outlines the experimental design used.

### 4.2. Isolation and In Vitro Culture of AD-MSCs

In a sterile setting, adipose tissue samples were collected from the inguinal region and washed with phosphate-buffered saline (PBS) (Cat. no. 09-8912-100, Medicago AB, Uppsala, Sweden). The tissue was then finely minced using sterile scissors in a 60 mm culture dish (Cat. no. TR4001, Nippon Genetics Co., Ltd., Tokyo, Japan) within a biosafety cabinet. These minced samples were placed in a water bath at 37 °C for one hour with gentle agitation. The bath contained Hank’s Balanced Salt Solution (HBSS) (Cat. no. 14025-092, Thermo Fisher Scientific Inc., New York, NY, USA) and 0.1% collagenase type I (Cat. no. 17100017, Gibco by Life Technologies, Waltham, MA, USA). To halt the collagenase’s activity, Dulbecco’s Modified Eagle’s Medium (DMEM) (Cat. no. 043-30085, FUJIFILM Wako Pure Chemical Corporation, Osaka, Japan), supplemented with 10% fetal bovine serum (FBS) (Cat. no. CCP-FBS-BR-500, COSMOBIO, Tokyo, Japan), was added.

Cell clusters were filtered out using a 100 μm filter (BD Falcon, Bedford, MA, USA). The cell suspension was then centrifuged at 800× *g* for 10 min, and the supernatant was discarded. The remaining cell pellets were resuspended and treated with 1 mL of red blood cell lysis buffer to remove red blood cells. After a 10-min incubation at 4 °C with the lysis buffer, the cells were washed with 10 mL of PBS. A second centrifugation at 600× *g* for 3 min was performed, the supernatant was removed, and the cell pellets were resuspended in DMEM containing 10% FBS, 1% non-essential amino acids (Cat. no. 139-15651, FUJIFILM Wako Pure Chemical Corporation, Osaka, Japan), and 1% Penicillin/Streptomycin (Cat. no. 161-23181, FUJIFILM Wako Pure Chemical Corporation, Osaka, Japan).

The AD-MSCs were cultured in a 100 mm dish (Cat. no. TR4002, Nippon Genetics Co., Ltd., Tokyo, Japan) and incubated at 37 °C in a humidified atmosphere with 5% CO_2_. Once the cells reached approximately 80% confluence, they were transferred to the next passage. This process was continued until passage 4.

### 4.3. Cryopreservation and Recovery of AD-MSCs

A total of 1 million cells from passage 4 were placed into 2 mL cryovials (Cat. no. FG-CRY-In-20S, Nippon Genetics Co., Tokyo, Japan) and stored at −80 °C in 1 mL of Bambanker solution (Cat. no. CS-02-001, GC LYMPHOTEC Inc., Tokyo, Japan). After a storage period of one month, the cryovials were removed and thawed in a water bath maintained at 37 °C. Once thawed, the cells were centrifuged to obtain cell pellets, which were then resuspended in DMEM containing 10% fetal bovine serum (FBS), 1% non-essential amino acids, and 1% Penicillin/Streptomycin. The cells were subsequently cultured and allowed to proliferate until they reached passage 4.

### 4.4. Cell Morphological and Immunophenotypic Characterization of AD-MSCs

The cell morphology of both groups (AD-MSCs BF.C. and AD-MSCs AF.C.) was examined using an inverted microscope (CKX31, Olympus, Tokyo, Japan) [27]. Additionally, immunophenotypic characterization was conducted using flow cytometry. MSC surface markers were identified with antibodies against CD29 (PE, eBioscience™, San Diego, CA, USA, Catalog # 12-0291-82) and CD90 (PE, eBioscience™, Catalog # 12-0900-81), while CD45 (PE, Catalog # MA5-17379) was used as a marker for hematopoietic cells. The isotype controls included Armenian Hamster IgG Isotype Control (eBio299Arm) (PE, eBioscience™, Catalog # 12-4888-81), Mouse IgG2a kappa Isotype Control (eBM2a) (PE, eBioscience™, Catalog # 12-4724-42), and Mouse IgG2a Isotype Control (PE, Catalog # MG2A04) [95,96]. For the flow cytometry analysis, cells were initially washed three times with HBSS and then adjusted to a concentration of 1 × 10^6^ cells/mL. The cell suspensions were incubated in the dark at 4 °C for 20 min with the designated antibodies, using the concentrations specified by the manufacturer. To remove any unbound antibodies, the cells were washed again with HBSS. Flow cytometry was carried out using a Beckman Coulter flow cytometer (Brea, CA, USA) to evaluate and quantify the expression of cell surface antigens. The data obtained were processed and analyzed using CytExpert Software version 2.3 (Beckman Coulter, Brea, CA, USA).

### 4.5. AD-MSCs’ Differentiation (Adipogenesis, Osteogenesis, and Chondrogenesis)

To determine the multipotency of AD-MSCs, their differentiation potential was evaluated. Passage 4 MSCs from the two groups (AD-MSCs BF.C. and AD-MSCs AF.C.) were exposed to specialized induction media to induce their differentiation into adipogenic, osteogenic, and chondrogenic lineages. The effectiveness of the differentiation was assessed by analyzing tissue-specific staining. The percentage of stained cells was quantified using ImageJ software (1.8.0-345), which can be accessed at https://imagej.nih.gov/ij/download.html (accessed on 22 July 2024). Specifically, this quantification was performed within a defined observation area to ensure consistent measurements across all samples.

#### 4.5.1. Adipogenic Induction

During the 4th passage, AD-MSCs were seeded in a 6-well plate at a density of 1 × 10^5^ cells per well. Once the cells reached 80–100% confluence, an adipogenic induction medium was applied. This medium comprised DMEM supplemented with 10% FBS, 1 μM dexamethasone (Cat. no. D4902, Sigma Aldrich, St Louis, MO, USA), 500 μM isobutylmethylxanthine (Cat. no. I5879, Sigma Aldrich, St Louis, MO, USA), 100 μM indomethacin (Cat. no. I7378, Sigma Aldrich, USA), and 5 μg/mL insulin (Cat. no. I5500, Sigma Aldrich, St Louis, MO, USA). Additionally, an uninduced culture medium (DMEM+10% FBS) was employed as a negative control. Media replacement occurred every three days across all wells. After 21 days, the cells were harvested, and Oil Red O staining (Cat. no. O-0625, Sigma-Aldrich, St. Louis, MO, USA) was employed to identify intracellular lipid accumulation. The presence of red-stained fat vacuoles indicated their successful differentiation into adipocytes.

#### 4.5.2. Osteogenic Induction

AD-MSCs at passage 4 were seeded in a 6-well plate at a density of 1 × 10^5^ cells per well. Once the cells reached 80–100% confluence, they were exposed to an osteogenic differentiation medium. This medium was composed of DMEM supplemented with 10% FBS, 100 nM dexamethasone, 0.2 mM ascorbic acid (Cat. no. 016-04805, FUJIFILM Wako Pure Chemical Corporation, Osaka, Japan), and 10 mM β-glycerophosphate (Cat. no. G9422, Sigma Aldrich, St. Louis, MO, USA). Control wells received an uninduced culture medium. The media were refreshed every three days for a total of 21 days. The cells’ osteogenesis differentiation capability was assessed using an Alizarin Red stain (ALZ) (Cat. no. 40-1009-5, Sigma-Aldrich, St. Louis, MO, USA), which visualized the mineralized matrix. The presence of red staining in the matrix indicated successful osteogenic differentiation.

#### 4.5.3. Chondrogenic Induction

Similarly, AD-MSCs at their fourth passage were seeded at a density of 1 × 10^5^ cells per well in a 6-well plate with a serum-free medium designed for chondrogenic differentiation (Cat. no. C-28012, PromoCell GmbH, Heidelberg, Germany). The medium was changed every three days over a 21-day period. To evaluate chondrogenesis, Alcian Blue staining (Cat. no. 66011, Sigma-Aldrich, St. Louis, MO, USA) was performed to visualize the highly sulfated proteoglycans in the cartilaginous matrices using a light microscope.

### 4.6. Gene Expression of Cultured AD-MSCs

With the same two groups (AD-MSCs BF.C. and AD-MSCs AF.C.), we used real-time quantitative PCR to evaluate the pluripotency and immunomodulatory markers within cultured MSCs before and after their cryopreservation. The procedure commenced with the extraction of total RNA using the FastGene RNA Basic Kit (Cat. no. FG-80250, Nippon Genetics Co., Tokyo, Japan), in accordance with the manufacturer’s protocol. To eliminate any genomic DNA contamination, the extracted RNA was processed with the TURBO DNA-free™ Kit (Cat. no. AM1907, Thermo Fisher Scientific Inc., New York, NY, USA). The concentration of RNA was determined using a NanoDrop™ Lite spectrophotometer. Subsequently, first-strand cDNA synthesis was carried out using the ReverTra Ace™ qPCR RT Master Mix (Cat. no. FSQ-201, TOYOBO, Osaka, Japan), following the manufacturer’s instructions.

The RT-qPCR reaction mix included 2 μL of cDNA, 0.5 μL each of forward and reverse primers (10 μmol/L), 10 μL of THUNDERBIRD™ Next SYBR™ qPCR Mix (Cat. no. QPX-201, TOYOBO, Osaka, Japan), and 7 μL of dH_2_O. The thermal cycling protocol included an initial denaturation step at 95 °C for 30 s. This was followed by 40 cycles, each consisting of 5 s at 95 °C for denaturation and 10 s at 60 °C for annealing and extension. To quantify gene expressions, the 2^−ΔΔCT^ method was employed, with beta-actin used as the reference for normalization.

The mRNA levels of the pluripotent marker reduced expression 1 (*REX1*) and immunomodulatory markers transforming growth factor beta 1 (*TGFβ1*) and interleukin 6 (*IL-6*) were validated using RT-qPCR. Table 1 provides information on the specific primers used.

### 4.7. Cardiomyogenic Differentiation

AD-MSCs at their 4th passage and belonging to two other groups (AD-MSCs BF.C, AF. Cardio. diff. and AD-MSCs AF.C, AF. Cardio. diff.) were exposed to 10 μmol of 5-Azacytidine (Catalog # A2385, Sigma-Aldrich) for 24 h [97,98]. After the treatment period, the cells were washed three times with 1xPBS and then maintained in basal media consisting of DMEM and 10% FBS. For comparison, AD-MSCs cultured solely in basal media without any treatment were used as a control group.

### 4.8. Morphological Alterations

Following their exposure to 5-Azacytidine, the cells were monitored for any shifts in morphology suggestive of cardiomyocyte differentiation, such as changes in cell shape, and the development of structures resembling myotubes. Any observed modifications were documented, and images were taken after 21 days of induction using an inverted microscope.

### 4.9. Cell Viability Assay

A week after induction, cell viability in the induced groups was examined (AD-MSCs BF.C, AF. Cardio. diff. and AD-MSCs AF.C, AF. Cardio. diff.). Cells were treated using the Live/Dead Cell Viability Assay Kit (Cat. no. ab287858) and incubated at 37 °C for 15 min, according to the manufacturer’s protocol. Subsequently, the cells were promptly examined under a fluorescence microscope (BZ-9000, KEYENCE Instruments Inc., Tokyo, Japan). Healthy cells were distinguished by a green stain from Cell Dye II/Live Cell Staining Dye, while dead cells were marked by a red stain [99,100]. The images were analyzed using ImageJ (available at https://imagej.nih.gov/ij/download.html) (accessed on 28 July 2024), which counted the cells. Cell viability was assessed by calculating the ratio of live cells to the total cell count.

### 4.10. Immunofluorescence Staining

Three weeks post-induction, immunofluorescence staining was conducted to assess the cardiomyocyte differentiation of AD-MSCs in the 5-Azacytidine-treated groups. This evaluation targeted specific cardiomyocyte proteins, including cTnT, α-Actinin, and MHC. The procedure involved removing the culturing medium and gently washing the cells with PBS. After fixation with 4% paraformaldehyde (Cat. no. 09154-85) at 4 °C for 15 min, the cells were washed and permeabilized with 0.5% Triton X-100(Cat. no.9036-19-5). Subsequently, they were blocked with 1% bovine serum albumin (BSA) (Cat. no. A9418-5G) for 30 min at room temperature. Following a 1-h incubation with primary antibodies against these cardiac-specific proteins at 20 °C, which included cTnT, α-Actinin, and MHC (Cat. no. ab209813; 1:400; Abcam, Cambridge, UK, Cat. no. 701914, 1:100, Invitrogen, Waltham, MA, USA, Cat. no. PA5-117077, 1:200, Invitrogen, Waltham, MA, USA, respectively) [101,102,103]. The cells were washed and incubated with secondary antibodies (Goat Anti-Rabbit IgG H&L (FITC), 1:1000, ab6717, Abcam, and Goat anti-Rabbit IgG (H+L) Cross-Adsorbed Secondary Antibody, FITC, 2 µg/mL, F-2765, Invitrogen) for 1 h at room temperature, while protected from direct light. After counterstaining the cell nuclei with 4′, 6-diamidino-2-phenylindole (DAPI) (DAPI, 5 μg/mL; 1:1000, Cat. no. D3571, Invitrogen) [104], the slides were examined using a fluorescence microscope BZ-9000 (KEYENCE Instruments Inc., Tokyo, Japan).

### 4.11. Quantitative Assessment of Cardiac-Specific Genes

Total RNA was extracted from the AD-MSCs groups (AD-MSCs BF.C, AF. Cardio. diff. and AD-MSCs AF.C, AF. Cardio. diff.) using the FastGene RNA Premium Kit (Nippon Genetics, Tokyo, Japan) following the manufacturer’s protocol, as described previously for the gene expression analysis. RNA’s quantity and purity (OD260/OD280 ≈ 2.0) were assessed with a NanoDrop 2000 ultra-micro spectrophotometer (Thermo Fisher Scientific, Cambridge, MA, USA). Subsequently, 1 μg of RNA was reverse-transcribed into cDNA using the PrimeScript RT reagent Kit (Takara Bio, Shiga, Japan), following the same procedures detailed earlier. The expression levels of cardiomyocyte-specific genes, including *Troponin I*, Myocyte enhancer factor 2C (*MEF2c*), and Glycogen synthase kinase-3 beta (*GSK-3β*), were quantified using the StepOnePlus™ Real-Time PCR System (Thermo Fisher Scientific, Waltham, MA, USA) and the specific primers listed in Table 1. Their relative quantification was carried out using the 2^−ΔΔCT^ method, with normalization against Beta-actin [105].

### 4.12. Statistical Analysis

Data are expressed as means ± standard deviation and analyzed using Student’s *t*-test and a one-way ANOVA. A *p*-value below 0.05 was deemed statistically significant. Statistical analyses were conducted using GraphPad Prism 8.0 (GraphPad Software, San Diego, CA, USA) (8.4.0 (671)).

## 5. Limitations

This study employed a single cryopreservation protocol and focused on AD-MSCs at passage 4 (P4), which may limit the generalizability of our findings. While we assessed gene expression using PCR, the absence of additional protein analyses, such as a Western Blot, represents a limitation in the full evaluation of the impact of cryopreservation on protein expression. Future studies should explore multiple cryopreservation techniques, different cell passages, and incorporate protein-level assessments to further validate and expand upon our results. Long-term studies are also necessary to determine the enduring functional integrity and therapeutic efficacy of cryopreserved AD-MSCs.

## 6. Conclusions

Cryopreservation is crucial for preserving adipose-derived mesenchymal stem cells (AD-MSCs) for clinical use. Our study reveals that it has significant effects on their biological characteristics and cardiomyogenic potential. While cryopreservation maintains cells’ structure, surface marker expression, and multilineage differentiation capability, it noticeably diminishes the expression levels of key markers such as *REX1*, *TGFβ1*, and *IL-6*. Following their differentiation into cardiomyocytes, cryopreserved AD-MSCs exhibit an attenuated upregulation of cardiac-specific genes (*Troponin I*, *MEF2c*, *GSK-3β*) compared to their non-cryopreserved counterparts, despite retaining their cell viability and expression of cardiac-specific proteins. These findings emphasize the need to refine the cryopreservation protocols specifically tailored to AD-MSCs to enhance their therapeutic potential in clinical applications.

## Figures and Tables

**Table 1:** Primer sequences.

Name		Direction	Primer Sequences (5′–3′)
Pluripotent marker gene	*REX1*	Forward	GCTCCGGCGGAATCGAGTGG
		Reverse	GCACGTGTTGCTTGGCGACC
Immunomodulatory marker genes	*TGFβ1*	Forward	ATGCCAACTTCTGTCTGGGG
		Reverse	GGTTGTAGAGGGCAAGGACC
	*IL-6*	Forward	CCACCCACAACAGACCAGTA
		Reverse	TCTGACAGTGCATCATCGCT
Cardiogenic marker genes	*Troponin I*	Forward	GAGCTTCAGGACCTATGCCG
		Reverse	GAGAGTGGGCCGCTTAAACT
	*MEF2c*	Forward	ATGCGGCTCTCTGAAGGATG
		Reverse	TAGCACACACACACACTGCA
	*GSK-3β*	Forward	GGATGATGGCCGAGACTCTG
		Reverse	AGGCTCCCTCCAAGATCCAT
Housekeeping gene	*Beta-actin*	Forward	CCCATCTATGAGGGTTACGC
		Reverse	TTTAATGTCACGCACGATTTC

## Data Availability

The data presented in this study are available on request from the corresponding author.
